# Supplementary material for: Analysis of PPARγ Signaling Activity in Psoriasis
Source: Int J Mol Sci. 2021 Aug 10;22(16):8603. doi: 10.3390/ijms22168603 (PMC8395241; doi:10.3390/ijms22168603)
Supplement: Supplementary file 1 [file ijms-22-08603-s001.zip › Supplemental materials_Analysis of PPARg signaling activity in psoriasis/Pathway models/Models images and html files/Model of PPARG related pathways in psoriasis (short version)/3835.html]

EGF


# Protein EGF

|  |  |
| --- | --- |
| URN | urn:agi-llid:1950 |
| Total Entities | 0 |
| Connectivity | 9325 |
| Name | EGF |
| Description | epidermal growth factor |
| Notes | This gene encodes a member of the epidermal growth factor superfamily. The encoded preproprotein is proteolytically processed to generate the 53-amino acid epidermal growth factor peptide. This protein acts a potent mitogenic factor that plays an important role in the growth, proliferation and differentiation of numerous cell types. This protein acts by binding with high affinity to the cell surface receptor, epidermal growth factor receptor. Defects in this gene are the cause of hypomagnesemia type 4. Dysregulation of this gene has been associated with the growth and progression of certain cancers. Alternative splicing results in multiple transcript variants, at least one of which encodes a preproprotein that is proteolytically processed. [provided by RefSeq, Jan 2016] |
| Primary Cell Localization | Extracellular |
| Class | Ligand |
| ObjectType | Protein |

---

|  |  |
| --- | --- |
| Pathway | Skin Aging |
|  | Prolactin Production |
|  | Vascular Endothelial Cell Activation by Growth Factors |
|  | Gastric and Pancreatic Lipase Function |
|  | G0/G1 Phase Transition |
|  | Proteins Involved in Atherosclerosis |
|  | Proteins Involved in Hypertrophic Cardiomyopathy |
|  | Proteins Involved in Arterial Hypertension |
|  | Smooth Muscle Cell Dysfunction in Pulmonary Hypertension |
|  | Proteins with Altered Expression in Melanoma |
|  | Melanoma |
|  | Proteins Involved in Melanoma |
|  | Proteins Involved in Psoriasis |
|  | Proteins Involved in Diabetic Nephropathy |
|  | Hepatic Stellate Cells in non-Alcoholic Fatty Liver Disease |
|  | Proteins Involved in Obesity |
|  | Proteins Involved in Prolactinoma |
|  | Prolactinoma Overiew |
|  | Cetuximab Resistance in Colorectal Cancer |
|  | Metastatic Colorectal Cancer |
|  | EGFR Nuclear Signaling in Colorectal Cancer |
|  | Proteins Involved in Ulcerative Colitis |
|  | Proteins Involved in Hepatocellular Carcinoma |
|  | Hepatocellular Carcinoma |
|  | Growth Factor Signaling in Hepatocellular Carcinoma |
|  | Proteins Involved in Pancreatic Cancer |
|  | Pancreatic Ductal Carcinoma |
|  | Growth Factor Signaling in Pancreatic Neoplasms |
|  | Pancreatic Neuroendocrine Tumors |
|  | Goblet-Cell Exocytosis in Asthma |
|  | Goblet-Cell Related Mucus Secretion in Asthma |
|  | Proteins Involved in Polycystic Ovary Syndrome |
|  | Estrogen Effects on Pregnancy Gingivitis |
|  | Proteins Involved in Autistic Disorder and Aggression |
|  | Glioblastoma, Primary |
|  | Proteins Involved in Glioblastoma |
|  | Glioblastoma, Secondary |
|  | Astrocytoma |
|  | Glioma Invasion Signaling |
|  | Proteins Involved in Glioma |
|  | Growth Factor Signaling in Neuroblastoma |
|  | Neuroblastoma |
|  | Proteins Involved in Neuroblastoma |
|  | Proteins Involved in Parkinson's Disease |
|  | Proteins Involved in Breast Cancer Related to ERBB2/VEGFR/Akt Signaling Pathway |
|  | ERBB/VEGFR/Akt Signaling in Breast Cancer |
|  | ESR1/ERBB Positive Luminal Breast Cancer |
|  | Breast Cancer |
|  | Endometrial Cancer |
|  | Endometrioid Endometrial Cancer |
|  | Proteins Involved in Endometrial Cancer |
|  | Clear Cell Endometrial Cancer and Papillary Serous Endometrial Cancer |
|  | Proteins Involved in Endometriosis |
|  | Endometrioid Ovarian Carcinoma |
|  | Proteins Involved in Endometrioid Ovarian Carcinoma |
|  | Proteins Involved in Mucinous Ovarian Carcinoma |
|  | EGFR and PTAFR Signaling in Ovarian Cancer |
|  | High-Grade Serous Ovarian Carcinoma |
|  | Low-Grade Serous Ovarian Carcinoma |
|  | Proteins Involved in Low-Grade Serous Ovarian Carcinoma |
|  | Ovarian Cancer |
|  | Mucinous Ovarian Carcinoma |
|  | Proteins Involved in Cholesteatoma |
|  | EGF/cyclinD1 Signaling in Acquired Middle Ear Cholesteatoma |
|  | Subepithelial Fibroblasts in Middle Ear Cholesteatoma |
|  | Cholesteatoma |
|  | HPV E5 Regulates EDNRA and EGFR Signaling Pathway in Keratinocytes |
|  | Androgen Receptor Coregulator ARA55 (TGFB1I1) in Prostate Cancer |
|  | Prostate Cancer |
|  | Proteins Involved in Prostate Cancer |
|  | Ca2+ Toxicity in Lens Cells |
|  | Cytokines Trigger Otitis Media |
|  | Proteins Involved in Chronic Obstructive Pulmonary Disease |
|  | Mucin Hyperproduction in Goblet and Mucous Cells |
|  | Proteins Involved in Chronic Bronchitis |
|  | Proteins Involved in Polycystic Kidney Disease |
|  | Tamoxifen Induced Endometrial Cancer |
|  | EGF -> CTNN Expression Targets |
|  | EGF -> HIF1A Expression Targets |
|  | EGF -> AP-1/ATF Expression Targets |
|  | EGF -> CREB/CREBBP/ELK/SRF/MYC Expression Targets |
|  | EGF -> MEF/MYOD/NFATC Expression Targets |
|  | EGF -> FOXO3A Expression Targets |
|  | EGF -> TP53 Expression Targets |
|  | EGF -> NCOR2 Expression Target |
|  | EGF -> STAT Expression Targets |
|  | EGFR -> NCOR2 Signaling |
|  | EGFR/ERBB3 -> MEF/MYOD/NFATC/MYOG Signaling |
|  | EGFR -> CTNND Signaling |
|  | EGFR/ERBB2 -> TP53 Signaling |
|  | EGFR/ERBB2 -> CTNNB Signaling |
|  | EGFR -> SMAD1 Signaling |
|  | EGFR/ERBB2 -> HIF1A Signaling |
|  | EGFR -> ZNF259 Signaling |
|  | EGFR -> AP-1/ATF2 Signaling |
|  | EGFR -> AP-1/CREB/ELK/SRF/MYC Signaling |
|  | EGFR/ERBB -> STAT Signaling |
|  | EREG -> EP300/SP1 Expression Targets |
|  | EGFR Transactivation in Cancer and Non-Cancer Cells |
|  | Receptors and Adaptor Proteins Activated in Cancer |
|  | Proteins with Altered Expression in Cancer-Associated Sustaining of Proliferative Signaling |
|  | G0/G1 Cell Cycle Phase Transition Activation in Cancer |
|  | STAT3 and NFkB Activate Inflammation-Induced Tumorigenesis |
|  | Proteins with Altered Expression in Tumor-Promoting Inflammation |
|  | VEGFA Dependent Angiogenesis in Cancer |
|  | Epithelial to Mesenchymal Transition in Cancer: Overview |
|  | Invadopodia Formation in Cancer Cells |
|  | Lipogenesis/Lipolysis Activation in Cancer Cells and Cancer-Associated Adipocytes |
|  | Proteins with Altered Expression in Cancer Metabolic Reprogramming |
|  | Tumor Infiltrating Macrophages in Cancer Progression and Immune Escape |
|  | FOXO1 Signaling |
|  | Estrogens/ESR1 non-Genomic Signaling |
|  | EGFR Signaling |
|  | ERK5/MAPK7 Signaling |
|  | Dioxin Induced Chloracne (Hypothesis) |
|  | prarg negative regulators, ps-positive |
|  | PPARG negative regulators |
|  | pparg expr target\_regulators |
|  | Neighbors of disease exacerbation |
|  | Neighbors of tumor progression |
|  | Neighbors of cancer progression |
|  | Neighbors of overall survival |
|  | Neighbors of disease severity |
|  | Neighbors of tumor size |
|  | Neighbors of lesion size |
|  | Neighbors of mortality |
|  | Neighbors of survival rate |
|  | Neighbors of infarct size |
|  | Neighbors of survival time |
|  | Neighbors of functional recovery |
|  | Neighbors of bacterial load |
|  | Neighbors of graft survival |
|  | Neighbors of therapeutic efficacy |
|  | Neighbors of clinical stage |
|  | Neighbors of lethality |
|  | Neighbors of tumor regression |
|  | Neighbors of disease-free survival |
|  | Neighbors of tumor response |
|  | Neighbors of long-term survival |
|  | Neighbors of treatment outcome |
|  | Neighbors of bone mass |
|  | Neighbors of body weight |
|  | Neighbors of body weight gain |
|  | Neighbors of microvessel density |
|  | Neighbors of tumor promotion |
|  | Neighbors of glucose tolerance |
|  | Neighbors of morbidity |
|  | Neighbors of fibrosis progression |
|  | Neighbors of mortality rate |
|  | Neighbors of fat mass |
|  | Neighbors of exhaustion |
|  | Neighbors of bone mineral density |
|  | Neighbors of adverse outcome |
|  | Neighbors of radioresistance |
|  | Neighbors of lesion area |
|  | Neighbors of cell density |
|  | Neighbors of progression-free survival |
|  | Neighbors of apoptosis rate |
|  | Neighbors of shear stress |
|  | Neighbors of viral load |
|  | Neighbors of blood pressure |
|  | Neighbors of tumor grade |
|  | Neighbors of vascular reactivity |
|  | Neighbors of cancer risk |
|  | Neighbors of tumor aggressiveness |
|  | Neighbors of blood glucose |
|  | Neighbors of renal hypertrophy |
|  | Neighbors of fasting plasma glucose |
|  | Neighbors of T-cell count |
|  | Neighbors of antimetastatic activity |
|  | Neighbors of glucose level |
|  | Neighbors of vascular tone |
|  | Neighbors of radiosensitivity |
|  | Neighbors of breast cancer risk |
|  | Neighbors of proliferation index |
|  | Neighbors of cell permeability |
|  | Neighbors of functional status |
|  | Neighbors of infarct area |
|  | Neighbors of fertility |
|  | Neighbors of pathological response |
|  | Neighbors of skin thickness |
|  | Neighbors of metabolic response |
|  | Neighbors of insulin level |
|  | Neighbors of fibroblast phenotype |
|  | Neighbors of disease exacerbation |
|  | Neighbors of tumor progression |
|  | Neighbors of cancer progression |
|  | Neighbors of overall survival |
|  | Neighbors of disease severity |
|  | Neighbors of tumor size |
|  | Neighbors of lesion size |
|  | Neighbors of mortality |
|  | Neighbors of survival rate |
|  | Neighbors of infarct size |
|  | Neighbors of survival time |
|  | Neighbors of functional recovery |
|  | Neighbors of bacterial load |
|  | Neighbors of graft survival |
|  | Neighbors of therapeutic efficacy |
|  | Neighbors of clinical stage |
|  | Neighbors of lethality |
|  | Neighbors of tumor regression |
|  | Neighbors of disease-free survival |
|  | Neighbors of tumor response |
|  | Neighbors of long-term survival |
|  | Neighbors of treatment outcome |
|  | Neighbors of bone mass |
|  | Neighbors of body weight |
|  | Neighbors of body weight gain |
|  | Neighbors of microvessel density |
|  | Neighbors of tumor promotion |
|  | Neighbors of glucose tolerance |
|  | Neighbors of morbidity |
|  | Neighbors of fibrosis progression |
|  | Neighbors of mortality rate |
|  | Neighbors of fat mass |
|  | Neighbors of exhaustion |
|  | Neighbors of bone mineral density |
|  | Neighbors of adverse outcome |
|  | Neighbors of radioresistance |
|  | Neighbors of lesion area |
|  | Neighbors of cell density |
|  | Neighbors of progression-free survival |
|  | Neighbors of apoptosis rate |
|  | Neighbors of shear stress |
|  | Neighbors of viral load |
|  | Neighbors of blood pressure |
|  | Neighbors of tumor grade |
|  | Neighbors of vascular reactivity |
|  | Neighbors of cancer risk |
|  | Neighbors of tumor aggressiveness |
|  | Neighbors of blood glucose |
|  | Neighbors of renal hypertrophy |
|  | Neighbors of fasting plasma glucose |
|  | Neighbors of T-cell count |
|  | Neighbors of antimetastatic activity |
|  | Neighbors of glucose level |
|  | Neighbors of vascular tone |
|  | Neighbors of radiosensitivity |
|  | Neighbors of breast cancer risk |
|  | Neighbors of proliferation index |
|  | Neighbors of cell permeability |
|  | Neighbors of functional status |
|  | Neighbors of infarct area |
|  | Neighbors of fertility |
|  | Neighbors of pathological response |
|  | Neighbors of skin thickness |
|  | Neighbors of metabolic response |
|  | Neighbors of insulin level |
|  | Neighbors of fibroblast phenotype |
|  | Neighbors of cell development |
|  | Neighbors of immune response |
|  | Neighbors of cell population |
|  | Neighbors of T-cell development |
|  | Neighbors of inflammatory response |
|  | Neighbors of wound healing |
|  | Neighbors of angiogenesis |
|  | Neighbors of innate immune response |
|  | Neighbors of cell function |
|  | Neighbors of macrophage activation |
|  | Neighbors of cell formation |
|  | Neighbors of osteoclast development |
|  | Neighbors of epithelial to mesenchymal transition |
|  | Neighbors of immunity |
|  | Neighbors of cell proliferative response |
|  | Neighbors of chemotaxis |
|  | Neighbors of dendritic cell differentiation |
|  | Neighbors of cell survival |
|  | Neighbors of osteoclast differentiation |
|  | Neighbors of cell phenotype |
|  | Neighbors of tissue remodeling |
|  | Neighbors of cell adhesion |
|  | Neighbors of T-cell activation |
|  | Neighbors of endothelial cell proliferation |
|  | Neighbors of fibrogenesis |
|  | Neighbors of tumor growth |
|  | Neighbors of dendritic cell development |
|  | Neighbors of tissue repair |
|  | Neighbors of cell count |
|  | Neighbors of cell infiltration |
|  | Neighbors of cell interaction |
|  | Neighbors of SMC proliferation |
|  | Neighbors of lumen formation |
|  | Neighbors of endothelial cell migration |
|  | Neighbors of adipogenesis |
|  | Neighbors of lipid storage |
|  | Neighbors of cell damage |
|  | Neighbors of pregnancy |
|  | Neighbors of aging |
|  | Neighbors of cell motility |
|  | Neighbors of bone resorption |
|  | Neighbors of fibroblast proliferation |
|  | Neighbors of adipocyte differentiation |
|  | Neighbors of macrophage migration |
|  | Neighbors of microglial activation |
|  | Neighbors of regeneration |
|  | Neighbors of cell migration |
|  | Neighbors of cancer growth |
|  | Neighbors of stem cell proliferation |
|  | Neighbors of smooth muscle cell migration |
|  | Neighbors of hepatic regeneration |
|  | Neighbors of transendothelial migration |
|  | Neighbors of phagocytosis |
|  | Neighbors of immune system activation |
|  | Neighbors of osteoclast formation |
|  | Neighbors of cell invasion |
|  | Neighbors of heart function |
|  | Neighbors of hemopoiesis |
|  | Neighbors of bone remodeling |
|  | Neighbors of ossification |
|  | Neighbors of T-cell function |
|  | Neighbors of immune system function |
|  | Neighbors of lung development |
|  | Neighbors of autophagy |
|  | Neighbors of monocyte adhesion |
|  | Neighbors of transcription activation |
|  | Neighbors of senescence |
|  | Neighbors of hepatocyte proliferation |
|  | Neighbors of cell transdifferentiation |
|  | Neighbors of neuroprotection |
|  | Neighbors of stem cell differentiation |
|  | Neighbors of endothelial cell function |
|  | Neighbors of neuronal death |
|  | Neighbors of cell growth |
|  | Neighbors of ROS generation |
|  | Neighbors of epithelial cell proliferation |
|  | Neighbors of first trimester pregnancy |
|  | Neighbors of cancer cell growth |
|  | Neighbors of sensitization |
|  | Neighbors of hemato-encephalic barrier |
|  | Neighbors of cellular senescence |
|  | Neighbors of colony formation |
|  | Neighbors of cell differentiation |
|  | Neighbors of keratinocyte proliferation |
|  | Neighbors of stem cell migration |
|  | Neighbors of life span |
|  | Neighbors of cell homeostasis |
|  | Neighbors of psoriasis |
|  | Neighbors of inflammatory disease |
|  | Neighbors of rheumatoid arthritis |
|  | Neighbors of atherosclerosis |
|  | Neighbors of fibrosis |
|  | Neighbors of colitis |
|  | Neighbors of arthritis |
|  | Neighbors of inflammatory bowel disease |
|  | Neighbors of injury |
|  | Neighbors of atherogenesis |
|  | Neighbors of vascular remodeling |
|  | Neighbors of metastasis |
|  | Neighbors of carcinogenesis |
|  | Neighbors of multiple sclerosis |
|  | Neighbors of chronic inflammation |
|  | Neighbors of inflammation |
|  | Neighbors of tumor microenvironment |
|  | Neighbors of pathological angiogenesis |
|  | Neighbors of obesity |
|  | Neighbors of vascular disease |
|  | Neighbors of diabetes mellitus |
|  | Neighbors of asthma |
|  | Neighbors of pneumonia |
|  | Neighbors of hyperplasia |
|  | Neighbors of liver fibrosis |
|  | Neighbors of chronic obstructive pulmonary disease |
|  | Neighbors of myocardial infarction |
|  | Neighbors of reperfusion injury |
|  | Neighbors of airway inflammation |
|  | Neighbors of hepatitis |
|  | Neighbors of infection |
|  | Neighbors of vascular injury |
|  | Neighbors of renal injury |
|  | Neighbors of preeclampsia |
|  | Neighbors of liver injury |
|  | Neighbors of neuroinflammation |
|  | Neighbors of sepsis |
|  | Neighbors of death |
|  | Neighbors of type 1 diabetes |
|  | Neighbors of encephalomyelitis |
|  | Neighbors of ischemic stroke |
|  | Neighbors of dermatitis |
|  | Neighbors of neutrophil infiltration |
|  | Neighbors of diabetic nephropathy |
|  | Neighbors of lung injury |
|  | Neighbors of immunopathology |
|  | Neighbors of systemic sclerosis |
|  | Neighbors of hepatocellular carcinoma |
|  | Neighbors of autoimmunity |
|  | Neighbors of acute lung injury |
|  | Neighbors of gastric cancer |
|  | Neighbors of atherosclerotic plaque |
|  | Neighbors of colorectal cancer |
|  | Neighbors of cardiac remodeling |
|  | Neighbors of breast cancer |
|  | Neighbors of ischemia |
|  | Neighbors of melanoma |
|  | Neighbors of cartilage degeneration |
|  | Neighbors of interstitial fibrosis |
|  | Neighbors of neoplasm invasion |
|  | Neighbors of kidney disease |
|  | Neighbors of lung disease |
|  | Neighbors of pulmonary fibrosis |
|  | Neighbors of cardiovascular disease |
|  | Neighbors of stroke |
|  | Neighbors of cancer |
|  | Neighbors of cerebral ischemia |
|  | Neighbors of type 2 diabetes |
|  | Neighbors of neoplasm |
|  | Neighbors of endometriosis |
|  | Neighbors of neointima |
|  | Neighbors of atrophy |
|  | Neighbors of myocardial fibrosis |
|  | Neighbors of endothelial cell dysfunction |
|  | Neighbors of prostate cancer |
|  | Neighbors of hypertrophy |
|  | Neighbors of ovarian cancer |
|  | Neighbors of pancreatic cancer |
|  | Neighbors of hypertension |
|  | Neighbors of epithelial cell |
|  | Neighbors of keratinocyte |
|  | Neighbors of vascular smooth muscle cell |
|  | Neighbors of umbilical vein endothelial cell |
|  | Neighbors of monocyte |
|  | Neighbors of endometrial stromal cell |
|  | Neighbors of alveolar macrophage |
|  | Neighbors of macrophage |
|  | Neighbors of adipocyte |
|  | Neighbors of fibroblast |
|  | Neighbors of airway epithelial cell |
|  | Neighbors of PBMC |
|  | Neighbors of stromal cell |
|  | Neighbors of airway smooth muscle cell |
|  | Neighbors of osteoblast |
|  | Neighbors of pneumocyte |
|  | Neighbors of endometrium cell |
|  | Neighbors of microglia |
|  | Neighbors of trophoblast |
|  | Neighbors of mononuclear cell |
|  | Neighbors of hepatic stellate cell |
|  | Neighbors of hepatocyte |
|  | Neighbors of decidual cell |
|  | Neighbors of kidney mesangial cell |
|  | Neighbors of endothelial cell |
|  | Neighbors of Kuppfer cell |
|  | Neighbors of proadipocyte |
|  | Neighbors of inflammatory cell |
|  | Neighbors of peritoneal macrophage |
|  | Neighbors of neutrophil |
|  | Neighbors of smooth muscle myocyte |
|  | Neighbors of astrocyte |
|  | Neighbors of mesothelial cell |
|  | Neighbors of mononuclear phagocyte |
|  | Neighbors of immunocompetent cell |
|  | Neighbors of granulocyte |
|  | Neighbors of cardiac myocyte |
|  | Neighbors of cancer-associated fibroblast |
|  | Neighbors of myeloid cell |
|  | Neighbors of mesenchymal stroma cell |
|  | Neighbors of corneal epithelial cell |
|  | Neighbors of cytotrophoblast |
|  | Neighbors of stem cell |
|  | Neighbors of tumor infiltrating macrophage |
|  | Neighbors of B-cell |
|  | Neighbors of granulosa cell |
|  | Neighbors of leukocyte |
|  | Neighbors of insulin-secreting cell |
|  | Neighbors of mesenchymal cell |
|  | Neighbors of T-cell |
|  | Neighbors of myofibroblast |
|  | Neighbors of pancreatic stellate cell |
|  | Neighbors of neuroglia |
|  | Neighbors of thyroid epithelial cell |
|  | Neighbors of mammary epithelial cell |
|  | Neighbors of phagocyte |
|  | Neighbors of kidney cell |
|  | Neighbors of lymphocyte |
|  | Neighbors of brain cell |
|  | Neighbors of M2 macrophage |
|  | Neighbors of retina cell |
|  | Neighbors of blood cell |
|  | Neighbors of lung |
|  | Neighbors of liver |
|  | Neighbors of kidney |
|  | Neighbors of skin |
|  | Neighbors of intestine |
|  | Neighbors of lymph node |
|  | Neighbors of colon |
|  | Neighbors of blood vessel |
|  | Neighbors of heart |
|  | Neighbors of gastrointestinal tract |
|  | Neighbors of bone |
|  | Neighbors of placenta |
|  | Neighbors of esophagus |
|  | Neighbors of retina |
|  | Neighbors of breast |
|  | Neighbors of stomach |
|  | Neighbors of artery |
|  | Neighbors of pancreas |
|  | Neighbors of prostate |
|  | Neighbors of decidua |
|  | Neighbors of amnion |
|  | Neighbors of vein |
|  | Neighbors of villus |
|  | Neighbors of bronchus |
|  | Neighbors of glands |
|  | Neighbors of cornea |
|  | Neighbors of lymph node of mesentery |
|  | Neighbors of mammary gland |
|  | Neighbors of brain blood vessel |
|  | Neighbors of bladder |
|  | Neighbors of blastocyst |
|  | Neighbors of salivary gland |
|  | Neighbors of endocrine system |
|  | Neighbors of NF-kB family |
|  | Neighbors of JNK |
|  | Neighbors of PKC |
|  | Neighbors of mitogen-activated protein kinase |
|  | Neighbors of transforming growth factor |
|  | Neighbors of cytokine |
|  | Neighbors of ovalbumin |
|  | Neighbors of SMAD subfamily |
|  | Neighbors of estrogen receptor |
|  | Neighbors of GPCR |
|  | Neighbors of Notch |
|  | Neighbors of lectin |
|  | Neighbors of endothelin |
|  | Neighbors of alpha2 adrenoceptor |
|  | Neighbors of gonadotropins |
|  | Neighbors of AHR subfamily |
|  | Neighbors of FSH |
|  | Neighbors of RA receptor |
|  | Neighbors of neuropeptides |
|  | Neighbors of histone acetyltransferase |
|  | Model of PPARG signaling in psoriasis |
|  | PPARG negative regulators and targets |
|  | Model of PPARG related pathways in psoriasis (short version) |
|  | 3\_Mesangial cell dysfunction in glomerulonephritis |
|  | 2\_Pathogens stimulate mucins expression in the middle ear |
|  | 2\_The intense proliferation and contractile activity of smooth muscle cells in pulmonary hypertension |
|  | 3\_1\_Mucus accumulation in asthma |
|  | 4\_Mucin hyperproduction in goblet and mucous cells in COPD |
|  | drug-target molsynthesis |
|  | Model of PPARG signaling in psoriais (tested) |

---

|  |  |
| --- | --- |
| Group | Chemokines, Growth Factors and Receptors Involved in Cancer Cells Chemotaxis |

---

|  |  |
| --- | --- |
| MedScan ID | 1950 |

---

|  |  |
| --- | --- |
| LocusLink ID | 1950 |
|  | 13645 |
|  | 25313 |

---

|  |  |
| --- | --- |
| Alias | epidermal growth factor urogastrone |
|  | AI790464 |
|  | urogastrone |
|  | epidermal growth factor (beta-urogastrone) |
|  | HOMG4 |
|  | beta-urogastrone |
|  | OTTHUMP00000219721 |
|  | epidermis growth factor |
|  | EGF |
|  | OTTHUMP00000219722 |
|  | LOC211904 |
|  | pro-epidermal growth factor |
|  | EGF\_MOUSE |
|  | EGF\_HUMAN |
|  | epidermis growth factor urogastrone |
|  | epidermal growth factor |
|  | URG |
|  | Pro-epidermal growth factor precursor (EGF) |

---

|  |  |
| --- | --- |
| GO ID | 0005088 |
|  | 0042813 |
|  | 0017147 |
|  | 0005509 |
|  | 0005154 |
|  | 0008083 |
|  | 0030297 |
|  | 0038128 |
|  | 0070371 |
|  | 0000165 |
|  | 0000187 |
|  | 0000186 |
|  | 0007171 |
|  | 0001525 |
|  | 0048754 |
|  | 0060070 |
|  | 0007173 |
|  | 0060749 |
|  | 0061024 |
|  | 1901185 |
|  | 0045746 |
|  | 0090370 |
|  | 0042059 |
|  | 0051048 |
|  | 0018108 |
|  | 0002576 |
|  | 0043388 |
|  | 0043406 |
|  | 0090263 |
|  | 0030335 |
|  | 0008284 |
|  | 0021940 |
|  | 0045741 |
|  | 0010628 |
|  | 1900127 |
|  | 0045840 |
|  | 0010800 |
|  | 0042327 |
|  | 0051897 |
|  | 1902966 |
|  | 0002092 |
|  | 0045893 |
|  | 2000060 |
|  | 0046425 |
|  | 0090279 |
|  | 2000145 |
|  | 2000008 |
|  | 0007165 |
|  | 0030665 |
|  | 0070062 |
|  | 0005576 |
|  | 0005615 |
|  | 0016021 |
|  | 0005765 |
|  | 0005886 |
|  | 0031093 |
|  | 0043235 |
|  | 2000573 |
|  | 0070374 |
|  | 0048146 |
|  | 0050731 |
|  | 0061098 |
|  | 0050730 |
|  | 0050708 |
|  | 0051223 |
|  | 0016020 |
|  | 0035556 |
|  | 0001822 |
|  | 0010469 |
|  | 0046934 |
|  | 0004713 |
|  | 0006260 |
|  | 0007262 |
|  | 0044332 |
|  | 0046854 |
|  | 0048015 |
|  | 0043547 |
|  | 0035413 |
|  | 0014066 |
|  | 0005622 |
|  | 0038095 |
|  | 0007596 |
|  | 0008543 |
|  | 0045087 |
|  | 0048011 |
|  | 0030168 |
|  | 0007243 |
|  | 0005625 |

---

|  |  |
| --- | --- |
| KEGG ID | hsa:1950 |
|  | mmu:13645 |
|  | rno:25313 |

---

|  |  |
| --- | --- |
| Organism | Homo sapiens {Organism urn:agi-taxid:9606} |
|  | Mus musculus {Organism urn:agi-taxid:10090} |
|  | Rattus norvegicus {Organism urn:agi-taxid:10116} |
|  | Homo sapiens |
|  | Mus musculus |
|  | Rattus norvegicus |

---

|  |  |
| --- | --- |
| Mouse chromosome position | 3 58.5 cM |
|  | 3 65.2 cM |

---

|  |  |
| --- | --- |
| OMIM ID | 131530 |
|  | 611718 |

---

|  |  |
| --- | --- |
| Rat chromosome position | 2q21 |
|  | 2q42-q43 |

---

|  |  |
| --- | --- |
| Hugo ID | 3229 |
|  | HGNC:3229 |

---

|  |  |
| --- | --- |
| Human chromosome position | 4q25 |

---

|  |  |
| --- | --- |
| Swiss-Prot Accession | P01133 |
|  | P01133.2 |
|  | A0A0G2JF92 |
|  | Q3UWD7 |
|  | A0A0G2JDT8 |
|  | P01132 |
|  | P01132.2 |
|  | P07522 |
|  | P07522.2 |
|  | B4DRK7 |
|  | E7EVD2 |
|  | E9PBF0 |
|  | Q52LZ6 |
|  | E9QNX6 |
|  | Q569W5 |
|  | Q6P9J2 |
|  | Q63183 |
|  | Q6QBS2 |
|  | Q8VD07 |
|  | Q6P6T8 |

---

|  |  |
| --- | --- |
| PIR ID | A25531 |
|  | I52995 |

---

|  |  |
| --- | --- |
| GenBank ID | NC\_000004 |
|  | XR\_001741157 |
|  | XR\_001741156 |
|  | NM\_001963 |
|  | NP\_001954 |
|  | NM\_001178130 |
|  | NP\_001171601 |
|  | NM\_001178131 |
|  | NP\_001171602 |
|  | NM\_001357021 |
|  | NP\_001343950 |
|  | XM\_017007845 |
|  | XP\_016863334 |
|  | XM\_017007846 |
|  | XP\_016863335 |
|  | XM\_017007850 |
|  | XP\_016863339 |
|  | XM\_017007847 |
|  | XP\_016863336 |
|  | XM\_017007849 |
|  | XP\_016863338 |
|  | XM\_017007851 |
|  | XP\_016863340 |
|  | XM\_017007848 |
|  | XP\_016863337 |
|  | XM\_005262796 |
|  | XP\_005262853 |
|  | XM\_017007853 |
|  | XP\_016863342 |
|  | XM\_017007855 |
|  | XP\_016863344 |
|  | XM\_017007854 |
|  | XP\_016863343 |
|  | XM\_011531707 |
|  | XP\_011530009 |
|  | NG\_011441 |
|  | AC004050 |
|  | AC005509 |
|  | AF023155 |
|  | AJ131611 |
|  | AY506357 |
|  | AAR84237 |
|  | CH471057 |
|  | EAX06257 |
|  | HF583660 |
|  | CCQ43157 |
|  | KF457809 |
|  | AI628974 |
|  | AK299306 |
|  | BAG61319 |
|  | AK307734 |
|  | AY548762 |
|  | AAS83395 |
|  | BC093731 |
|  | AAH93731 |
|  | BC113461 |
|  | AAI13462 |
|  | BC143357 |
|  | CA437074 |
|  | CU446298 |
|  | DB227969 |
|  | DB349864 |
|  | JQ346088 |
|  | AFA26280 |
|  | X04571 |
|  | CAA28240 |
|  | P01133 |
|  | NC\_000069 |
|  | XM\_011240011 |
|  | XP\_011238313 |
|  | XM\_011240010 |
|  | XP\_011238312 |
|  | NM\_010113 |
|  | NP\_034243 |
|  | NM\_001310737 |
|  | NP\_001297666 |
|  | XM\_006500990 |
|  | XP\_006501053 |
|  | XR\_001783650 |
|  | NM\_001329594 |
|  | AC098732 |
|  | CH466532 |
|  | EDL12237 |
|  | EDL12238 |
|  | U69534 |
|  | X08047 |
|  | AK084927 |
|  | AK085707 |
|  | AK088408 |
|  | AK136439 |
|  | BAE22978 |
|  | AK142666 |
|  | AK157778 |
|  | BB635280 |
|  | BC017681 |
|  | AAH17681 |
|  | BC060741 |
|  | AAH60741 |
|  | BC092277 |
|  | AAH92277 |
|  | BP761200 |
|  | J00380 |
|  | AAA37539 |
|  | V00741 |
|  | CAA24115 |
|  | X74038 |
|  | P01132 |
|  | NC\_005101 |
|  | NM\_012842 |
|  | NP\_036974 |
|  | AC\_000070 |
|  | AABR07008655 |
|  | AABR07008656 |
|  | AABR07008657 |
|  | AABR07008658 |
|  | AAHX01020327 |
|  | AAHX01020328 |
|  | AAHX01020329 |
|  | AAHX01020330 |
|  | AF029212 |
|  | CH473952 |
|  | EDL82179 |
|  | EDL82180 |
|  | BC062030 |
|  | AAH62030 |
|  | JX149564 |
|  | JX437943 |
|  | AFR51946 |
|  | U04842 |
|  | AAB60436 |
|  | P07522 |
|  | XM\_006714124 |
|  | XP\_006714187 |
|  | XM\_005262798 |
|  | XP\_005262855 |
|  | XM\_005262800 |
|  | XP\_005262857 |
|  | XM\_005262801 |
|  | XP\_005262858 |
|  | XM\_005262797 |
|  | XP\_005262854 |
|  | XR\_427532 |
|  | XR\_938699 |
|  | XM\_011531708 |
|  | XP\_011530010 |
|  | XM\_005262802 |
|  | XP\_005262859 |
|  | XM\_011531709 |
|  | XP\_011530011 |
|  | NC\_018915 |
|  | AMYH02009902 |
|  | J02548 |
|  | AAA72173 |
|  | XM\_006500991 |
|  | XP\_006501054 |
|  | XR\_867211 |
|  | AC\_000025 |
|  | AAHY01031870 |
|  | AAHY01031871 |
|  | AAHY01031872 |
|  | AAHY01031873 |
|  | AAHY01031874 |
|  | AABR07013390 |
|  | AABR07013391 |
|  | AABR07013393 |
|  | AABR07013394 |
|  | AABR07013395 |
|  | AABR07013396 |
|  | AABR07013397 |
|  | AABR07013398 |
|  | AABR07013399 |
|  | AABR07072711 |
|  | XM\_006714125 |
|  | XP\_006714188 |
|  | XM\_005262799 |
|  | XP\_005262856 |
|  | AC\_000136 |
|  | ABBA01063785 |
|  | ABBA01063786 |
|  | XM\_006500992 |
|  | XP\_006501055 |
|  | XR\_375487 |
|  | XM\_006232008 |
|  | XP\_006232070 |
|  | XM\_006232009 |
|  | XP\_006232071 |
|  | AABR06013300 |
|  | AABR06013732 |
|  | AABR06013733 |
|  | AABR06013734 |
|  | AABR06013735 |
|  | AABR06013736 |
|  | AABR06020686 |
|  | XM\_003749222 |
|  | XP\_003749270 |
|  | NT\_016354 |
|  | NW\_001838915 |
|  | NW\_922162 |
|  | AC\_000047 |
|  | Q6QBS2 |
|  | NT\_039240 |
|  | NW\_001030737 |
|  | Q3UWD7 |
|  | Q569W5 |
|  | Q8VD07 |
|  | NW\_047627 |
|  | NW\_001084808 |
|  | Q6P6T8 |

---

|  |  |
| --- | --- |
| Swiss-Prot ID | EGF\_HUMAN |
|  | EGF\_MOUSE |
|  | EGF\_RAT |
|  | B4DRK7\_HUMAN |
|  | E9QNX6\_MOUSE |
|  | E7EVD2\_HUMAN |
|  | E9PBF0\_HUMAN |

---

|  |  |
| --- | --- |
| Cell Localization | Membrane |
|  | Extracellular |

---

|  |  |
| --- | --- |
| Ensembl ID | ENSG00000138798 |
|  | ENSP00000421384.1 |
|  | ENST00000503392.1 |
|  | ENSP00000424316.1 |
|  | ENST00000509793.5 |
|  | ENSP00000498337.1 |
|  | ENST00000652245.1 |
|  | ENSP00000265171.5 |
|  | ENST00000265171.9 |
|  | ENSMUSG00000028017 |
|  | ENSMUSP00000143075.1 |
|  | ENSMUST00000197079.4 |
|  | ENSMUSP00000029653.2 |
|  | ENSMUST00000029653.6 |
|  | ENSRNOG00000053979 |
|  | ENSRNOP00000074125.1 |
|  | ENSRNOT00000083713.1 |
|  | ENSP00000421384 |
|  | ENST00000503392 |
|  | ENSP00000424316 |
|  | ENST00000509793 |
|  | ENSP00000265171 |
|  | ENST00000265171 |
|  | ENSMUSP00000029653 |
|  | ENSMUST00000029653 |
|  | ENSMUSP00000143075 |
|  | ENSMUST00000197079 |

---

|  |  |
| --- | --- |
| MGI ID | MGI:95290 |
|  | 95290 |

---

|  |  |
| --- | --- |
| RGD ID | 2542 |

---

|  |  |
| --- | --- |
| Unigene ID | Hs.419815 |
|  | Mm.252481 |
|  | Rn.6075 |

---

|  |  |
| --- | --- |
| Homologene ID | 1483 |

---

|  |  |
| --- | --- |
| Shape | Rhomb |

---

|  |  |
| --- | --- |
| IPI ID | IPI00231848 |
|  | IPI00000073 |
|  | IPI00966866 |
|  | IPI00132121 |
|  | IPI00890244 |
|  | IPI00967121 |

---
